# Clinical Prospective Assessment of Genotoxic and Cytotoxic Effects of Fluoride Toothpaste and Mouthwash in Buccal Mucosal Cells

**DOI:** 10.3390/biomedicines10092206

**Published:** 2022-09-06

**Authors:** Ema Puizina Mladinic, Jasna Puizina, Lidia Gavic, Antonija Tadin

**Affiliations:** 1 Department of Maxillofacial Surgery, Clinical Hospital Centre Split, 21000 Split, Croatia; 2 School of Medicine, University of Split, 21000 Split, Croatia; 3 Department of Biology, Faculty of Science, University of Split, 21000 Split, Croatia; 4 Department of Restorative Dental Medicine and Endodontics, Study of Dental Medicine, School of Medicine, University of Split, 21000 Split, Croatia

**Keywords:** fluoride, mouthwash, micronucleus test, oral hygiene products, toothpaste, buccal mucosa, DNA damage

## Abstract

Background: Fluorides are an essential component of oral hygiene products used to prevent dental decay. Therefore, a question arises about the potential harms of joint use of fluoridated toothpaste and mouthwashes regarding the increased amount of fluoride in the oral mucosa. Methods: This prospective, double-blinded parallel randomized clinical trial was conducted using a buccal micronucleus cytome assay (BMCyt assay). Forty-one participants were randomly assigned to the two groups. All participants used the same kinds of toothpaste for 12 weeks, designed explicitly for this study (non-fluoride, 1050 ppm F, and 1450 ppm F each for 4 weeks). Simultaneously, during the 3 months of the research, one group used mouthwash with fluoride (450 ppm) and another without fluoride. The buccal mucosal sampling was taken before using the tested products and after 4, 8, and 12 weeks of their use. Results: The frequency of micronuclei and the majority of other scored endpoints from the BMCyt assay showed no statistically significant differences within and between the studied groups. Comparing two groups, only statistically significant increases in the number of cells with nuclear buds (*p* = 0.048) and karyorrhexis (*p* = 0.020) at four weeks of usage were observed in the group that used mouthwash with fluoride. Conclusion: On the basis of the results, it can be concluded that simultaneous application of fluoridated toothpaste and fluoride mouthwash does not lead to cytogenetic damage in buccal mucosal cells.

## 1. Introduction

Oral hygiene products, including toothpaste and mouthwashes, are classified as cosmetic products which do not have to pass as strict risk assessment testing as drugs [1]. Fluoride is the most important component of oral hygiene products since it is widely used for the prevention of the dental decay in different forms—toothpaste, mouthwashes, varnishes, and gels. The safety of fluoride remains a controversial issue, not only regarding dental and skeletal fluorosis but also the fact that different studies have indicated significant genotoxic effects both in vivo and in vitro [2,3,4]. The evidence suggests that low-fluoride (<600 ppm F) toothpaste provides less caries protection than standard (1000 ppm F) or high-concentration (1500 ppm F) formulations [5].

Although there have been many epidemiological, pathogenetic, clinical, and cytogenetic studies associated with fluoride toxicity [6,7,8], the cytotoxic mechanism of fluoride in periodontal tissue is still not completely understood. Using fluoride dentifrices increases fluoride concentration in saliva and dental plaque, but also penetrates epithelial cells [9]. The buccal mucosa provides a barrier to potential carcinogens that can be metabolized to generate potential reactive products. As up to 90% of all cancers appear to be epithelial in origin, the buccal mucosa could be used to monitor early genotoxic events [10]. Ceppi et al. [11] found a strong correlation of genotoxic damage in buccal exfoliated cells with that in lymphocytes, which implies that systemic genotoxic effects within the bloodstream may also impact and be detectable in buccal cells. Furthermore, findings on exposure and genetic variables affecting micronucleus (MN) frequency in lymphocytes may potentially apply to some degree to buccal cells, including the association of MN with cancer risk [12]. This pathobiological event has several biological implications because genetic damage is closely involved in several diseases including cancer [13].

The buccal mucosa is a stratified squamous epithelium consisting of four distinct layers and represents an easily accessible tissue that can be used to sample cells in a minimally invasive manner, and it has been utilized to study the rate of division of proliferating (basal) cells, their genomic stability, and their propensity for cell death [14,15]. It is estimated that the permeability of the buccal mucosa is 4–4000 times greater than that of the skin since the mucosa is well supplied with both vascular and lymphatic drainage, while first-pass metabolism in the liver and pre-systemic elimination in the gastrointestinal tract are avoided [16].

Over the last decade, the buccal micronucleus cytome assay (BMCyt assay) emerged as a very important biomonitoring tool for detecting damage to genetic material in humans. The major advantages of the assay are its simplicity and easily accessible tissue. It allows the analysis of several parameters such as DNA and chromatin damage, cell death, and cytokinesis defects [10,17].

Fluoride applies its caries-controlling effect on the dental hard tissues during bacteria-produced acid challenges, while intraoral fluoride reservoirs serve to maintain elevated salivary fluoride levels. Fluoride reservoirs in the oral cavity may consist of the oral mucosa, calculus, dental biofilm, and caries lesions [18]. Rose et al. [19] suggested that the fluoride in the oral mucosa is bound to epithelial cell surfaces. Considering the strong evidence for a relationship between genetic damage and carcinogenesis, elucidation of the mechanisms of genotoxicity induced by fluoride is important to measure the degree of risk involved as far as to mitigate potential risks to human populations [13,20].

To date, numerous in vitro studies have analyzed the effects of fluoride on various mammalian cells such as oral cells (odontoblasts, ameloblasts, and gingival fibroblasts), bone cells (osteoblasts), blood cells (myeloma peripheral blood cells and leukemia cells), and brain, reproductive, and liver cells [21], in addition to in vivo studies on oral [1,22,23], bone, blood, liver, spleen, reproductive system, central nervous system, and kidney cells [4]. However, no in vivo studies on human regarding the genotoxicity of the joint use of fluoride toothpaste and mouthwash on buccal mucosal cells have been performed in this way [24].

Lifelong everyday use of oral health products has increased [25]; therefore, a question arises about the potentially harmful impact of using more than one fluoride product every day regarding the amount of fluoride on the oral mucosa. Since there is no limitation in the usage of oral hygiene products and it is well known that the oral mucosal tissue can serve as a long-term fluoride reservoir [26], potential adverse effects should be verified by an appropriate in vivo study.

This study evaluated the cytotoxic and genotoxic effects of toothpastes with different fluoride concentrations (0 ppm F, 1050 ppm F, and 1450 ppm F) combined with the simultaneous application of fluoride mouthwash (450 ppm F) and mouthwash without fluoride. The exfoliated buccal mucosal cells were stained and microscopically evaluated for nuclear/cellular anomalies according to the buccal micronucleus cytome assay (BMCyt assay). The study’s null hypothesis was that there would be no significant difference in the number of cytogenetic damages between the group that used fluoride-free mouthwash and the group that used 450 ppm fluoride mouthwash.

## 2. Material and Methods

### 2.1. Study Design

This double-blinded, randomized controlled trial with two parallel groups evaluated toothpaste and mouthwash toxicity depending on the applied fluorine concentration. The aim was to assess the frequency of biomarkers indicative of DNA damage (micronuclei and nuclear buds), cellular proliferation potential (binucleated cells), and cell death (condensed chromatin, karyorrhexis, pyknosis, and karyolysis).

**Figure 1:**
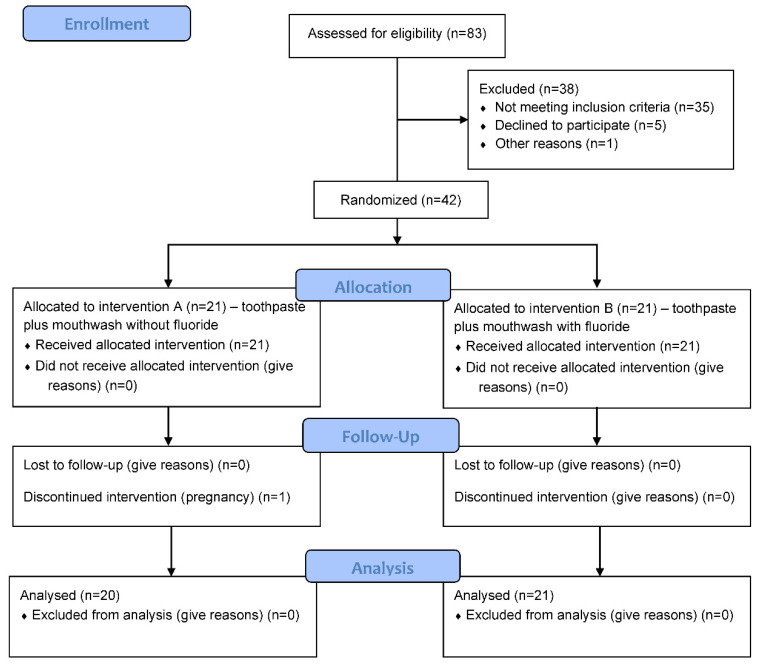
Flowchart of participant recruitment and follow-up.

The study was conducted at the Department of Restorative Dental Medicine and Endodontics, Study of Dental Medicine, School of Medicine, the University of Split, from March to October 2021. The study protocol was reviewed and approved by the Ethics Committee of the School of Medicine, the University of Split (No: 2181-198-03-04-20-0103). Furthermore, it was conducted following the Consolidated Standards of Reporting Trials (CONSORT) guidelines (Figure 1) [27] and registered at clinical trials (ClinicalTrials.gov, study ID number: NCT04801576). Participation was voluntary, and signed informed consent was obtained from all participants after explaining the purpose of the study. All the data were anonymized and treated confidentially according to current Croatian legislation on the treatment of sensitive data.

### 2.2. Participants and Sample Size

Participants were sampled among the School of Medicine University of Split and Clinical Hospital Center Split employees and students. Eighty-three students and employees were screened by routine dental examination, and 42 participants were finally recruited for the study according to the given inclusion and exclusion criteria. Inclusion criteria were non-smokers aged between 18 and 65 years, with good general health (ASA I physical status). In contrast, exclusion criteria were history of using antibiotics, corticosteroids, and anti-inflammatory drugs, history of radiation therapy in the head and neck region in the last 6 months, patients with any oral diseases, pregnancy, breast-feeding, prosthetic, orthodontic, and implant-supported rehabilitation, amalgam filling, or history of allergy to any dental hygiene product.

A detailed medical and dental anamnesis was taken from each participant. In addition, in a structured questionnaire tailored to this study, all participants provided answers to questions related to demographic factors (age, gender), personal factors (general health, a medication used, number of composite fillings, and radiation exposure), lifestyle (smoking, alcohol consumption, and exercise), eating habits, and oral hygiene habits.

The effect of sample size (Cohen’s d) obtained from the in vivo evaluation of fluoride and sodium lauryl sulfate in toothpaste on buccal epithelial cell toxicity [1] was used to calculate the minimum required sample size. From the difference in the number of occurrences of micronuclei in oral mucosal cells after using fluoride-free paste (0.55 ± 0.51) and after using fluoride paste (1.15 ± 0.88), the obtained sample size effect (Cohen’s d) was 0.835. Therefore, with a significance level of α = 0.05, 80% of the strength of the test, and the stated impact of sample size, the required sample size was 19 participants per group. 

### 2.3. Materials and Clinical Procedure

One month before the study began, all subjects used the same toothpaste without fluoride, polyethene glycol, and sodium lauryl sulfate (Biomed Calcimax, Splat, Moscow, Russia) in a preparation period. In addition, baseline buccal mucosa sampling was obtained from all participants to observe a possible difference in the number of cytogenetic impairments depending on demographic and social factors. 

Following the baseline sampling, eligible participants were randomly assigned into two groups (*n* = 21) following a computer software block randomization procedure [28]. This method was used to ensure balance in sample size across groups, and randomization was performed by a research member who was not part of the evaluation procedures. The participant code and toothpaste/mouthwash code were recorded in a chart for further reference.

Participants in both groups over the next 12 weeks (4 weeks each) used three different types of toothpaste: non-fluoride, toothpaste with 1050 ppm fluoride, and toothpaste with 1450 ppm fluoride, in the order stated. In addition, participants simultaneously used mouthwash: the first group without (Group A—0 ppm F) and the second group with fluoride (Group B—450 ppm F). Participants in group A first used fluoride-free toothpaste and fluoride-free mouthwash for 4 weeks. Immediately afterward, from the fifth to the eighth week of study, they continued to use toothpaste with 1050 ppm fluoride and mouthwash without fluoride. Subsequently, between the ninth and 12th week, participants in Group A used toothpaste with 1450 ppm fluoride and mouthwash without fluoride. Group B participants used the same kinds of toothpaste at the same time intervals as those in group A; however, they used fluoride mouthwash (450 ppm F) all stages.

Participants were instructed in writing to apply the tested toothpaste twice a day for 3 min in the amount of 1 g (2 cm) using a Bass brushing technique. Next, they used 5 mL of mouthwash, swishing it in their mouth for 30 s. All participants used the same soft toothbrush during the research (Colgate Slim Soft, Colgate—Palmolive Company, New York, NY, USA). They were also instructed to use nonfluorinated dental floss and refrain from using any other fluoride-containing products. The participants were free to stop participating in the trial whenever they wanted.

The composition of toothpaste and mouthwash was designed in collaboration with the pharmaceutical company Pharmagal (Split, Croatia). Ingredients for different kinds of toothpaste and mouthwash are presented in Table 1. All toothpaste tubes and mouthwash bottles were designed similarly. To ensure the blinding of participants and the principal investigator, different kinds of toothpaste were coded by numbers (1, 2 or 3), while mouthwash bottles were coded by letters (A or B). That procedure was performed by a pharmacist who was not involved in the random allocation process.

### 2.4. Buccal Mucosal Cell Sampling and Buccal Micronucleus Cytome Assay (BMCyt Assay)

Buccal mucosal cell sampling in both groups was performed at baseline (T0) and after 4 (T1), 8 (T2), and 12 weeks (T3) of using the tested toothpaste and mouthwash. At all sampling timepoints, samples were collected in the morning. One hour before the sampling, the participants were asked to abstain from consuming any food and drinks. They were also asked to rinse the oral cavity well with tap water immediately before taking the sample to remove the oral microflora and exfoliated cells. Swabs were taken by gently brushing the buccal mucosa bilaterally in a circular motion using a cytobrush (Cytobrush Plus, GmbH, Dietramszell-Linden, Germany).

The brushes were then dipped into tubes containing buccal cell buffer (1.6 g/L Tris-HCl, 38.0 g/L EDTA, and 1.2 g/L sodium chloride, pH 7.0) and repeatedly rotated to dislodge and release the cells into the buffer. Lastly, all samples were coded and transported to the molecular laboratory (Laboratory for Molecular Genetics, Faculty of Science, University of Split, Split).

The BMCyt assay was performed according to the procedure described by Thomas and Fenech [10]. For each subject, two slides were prepared by smearing 100 μL of cell suspension onto precleaned slides (approximately 1 × 10^5^ cells/slide). The cells were then stained by applying the Feulgen plus Fast Green method. Briefly, the slides were fixed in ethanol, 1 min each in 50% and 20% ethanol, washed with distilled water, and treated in 5 M HCl for 30 min. After washing in distilled water, the slides were drained and then stained with Schiff’s reagent for 60 min. The slides were further washed with distilled water and then counterstained with 0.2% Fast Green for 20 s. Air-dried slides were finally mounted with DePex mounting medium.

Schiff’s reagent and Fast green dye, as well as conventional microscope slides and coverslips, were supplied by BIOGNOST (Biognost d.o.o., Zagreb, Croatia). All other reagents used (ethanol, acetic acid, and phosphate-buffered saline (PBS)) were analytical grade.

**Figure 2:**
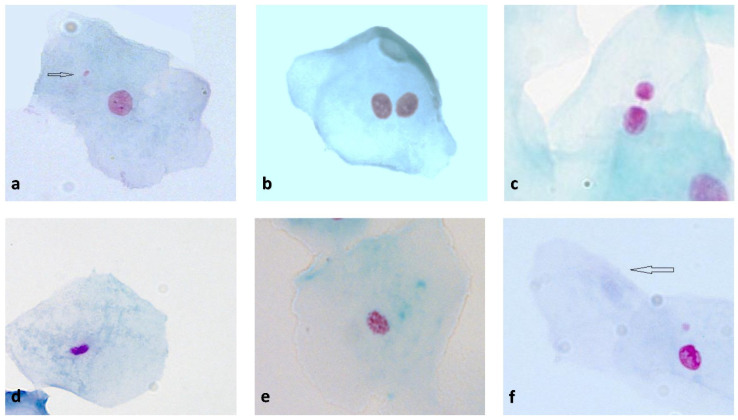
Images of the typical cell types scored in BMCyt assay, stained using Feulgen and Light Green, and viewed under transmitted light, all taken at ×1000 magnification: (**a**) cell with micronucleus (arrow points at micronucleus); (**b**) binucleated cell; (**c**) cell with a nuclear bud; (**d**) pyknotic cell; (**e**) karyorrhectic cell; (**f**) karyolytic cell (arrow points karyolytic cell).

The coded slides were read blind by following the scoring scheme proposed by the Thomas and Fenech Nature protocol [10]. Slides were examined at 1000× magnification using a bright-field microscope (Zeiss Axioimager M1, Karl Zeiss, Vienna, Austria) equipped with a high-resolution CCD camera (Carl Zeiss AxioCam MR Rev3) with Axio Vision Rel. 4.7 software (Karl Zeiss, Vienna, Austria). The slide preparations were scored to determine the frequency of anomalies associated with cell death, and nuclear abnormalities indicative of chromosomal instability or DNA damage were classified essentially according to established HUMNxl criteria [29] in a minimum of 1000 cells. Binucleated cells (BNCs) indicated a cytokinesis defect (cytotoxicity); condensed chromatin (CCC), karyorrhectic (KHC), pyknotic (PYK), and karyolytic (KYL) cells were regarded as markers of early-to-late stages of apoptosis and cell death [30]. The slides were then scored for cells with MN and nuclear buds (NBUDs) among a minimum of 2000 differentiated cells (1000/slide) as respective chromosomal and DNA damage measures. The photographic images in Figure 2 describe the classification of cells scored in the buccal cytome assay.

### 2.5. Statistical Analysis

Statistical analysis was performed using the SPSS software package (IBM Corp., Armonk, NY, USA). The distribution of variables was tested with the Kolmogorov–Smirnov test.

The primary statistical parameters (mean, standard deviation, minimum, maximum, median, and interquartile range values) were determined using descriptive statistical analysis. The differences in demographic data of respondents were tested using an independent *t*-test. 

The differences in the number of micronuclei and other nuclear anomalies between different sampling times for each group were tested using the Kruskal–Wallis test. The Mann–Whitney U test tested the differences in the number of micronuclei and other nuclear abnormalities between groups at the same sampling time. A general regression model (GRM) from the linear/nonlinear modeling method was used for the assessment of the influence of the predictor variables (age, gender, and dietary habits) on dependent variables (number of micronuclei, number of binucleated cells, nuclear buds, pyknosis, condensed chromatin, karyolysis, and karyorrhexis). The results of GRM were expressed in the form of Pareto charts of *t*-values. The significance level was set at 0.05 for all tests.

## 3. Results

Forty-one participants with a mean age of 35.00 ± 11.79 years were included in the study. The basic demographic and dental status characteristics of the participants in the two study groups are presented in Table 2. There were no significant differences between the two groups in terms of weight, height, gender, age, or dental status regarding composite filling.

The results of the buccal micronucleus cytome assay as cell proliferation markers (BNC), chromosomal and DNA damage markers (MN and NBUD), and cell death/apoptosis markers (CCC, KHC, PYK, and KYL), are presented in Table 3 and Table 4. According to the Mann–Whitney U test, the only significant differences, comparing two groups at the same sampling time, were observed in the number of cells with nuclear buds at timepoint T1 (*p* = 0.048) and karyorrhexis at timepoints T1 (*p* = 0.020) and T3 (*p* = 0.003).

According to the Kruskal–Wallis test, a statistically significant difference was observed in the number of binucleated cells and cells with condensed chromatin in both groups (*p* ≤ 0.001). The number of binucleated cells in group A and group B significantly decreased from the second—T2 to third—T3 timepoint (*p* = 0.020 and *p* = 0.008, respectively).

**Figure 3:**
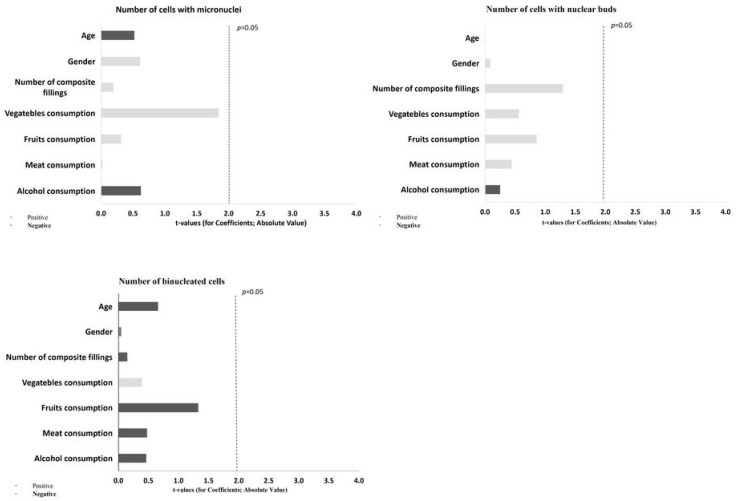
Multiple regression analysis results. Significant association of cytogenetic endpoints in buccal mucosal cells (number of cells with micronuclei, nuclear buds, and binucleated cells) with demographic and lifestyle factors as possible predictors.

**Figure 4:**
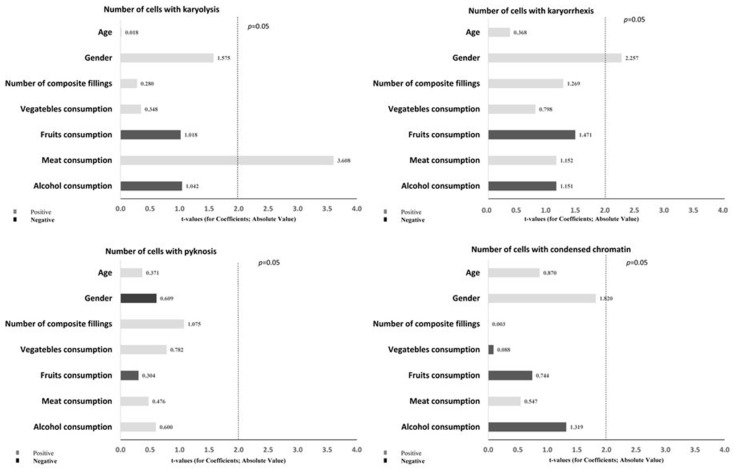
Multiple regression analysis results. Significant association of cytogenetic endpoints in buccal mucosal cells (number of cells with karyolysis, karyorrhexis, pyknosis, and condensed chromatin) with demographic and lifestyle factors as possible predictors.

The dependence of the cytogenetic damage score on all predictor variables was determined by multiple regression analysis and presented in the form of a Pareto diagram (Figure 3 and Figure 4). The number of cells with karyolysis (KYL) was statistically significantly affected by meat consumption (β = 41.263, SE = 11.437, *p* = 0.001), while the number of cells with karyorrhexis was statistically significantly affected by gender (β = 27.493; SE = 12.182, *p* = 0.032).

## 4. Discussion

In the last few years, a considerable number of published articles have demonstrated extensive evidence related to the genotoxicity and cytotoxicity of fluoride. Most of these studies were performed in vitro on a variety of human and animal cell lines, while some were conducted in vivo on experimental animals such as rats. Due to the scarcity of clinical in vivo studies, the toxicity of fluoride remains controversial [13,31].

This clinical in vivo study determined whether the joint and regular use of fluoridated oral hygiene products such as toothpaste and mouthwash may represent a hazard to human health as a consequence of excess fluoride in the human oral cavity. To the best of our knowledge, there are no such studies published yet. To address this question, we performed a buccal cytome assay on exfoliated buccal cells from 41 examinees. The research hypothesis was that there would be no difference within the groups depending on the concentration of the used products or between the groups depending on whether the participants used fluoride mouthwash or not. Our results showed that, for most of the observed parameters from the BMCyt assay, no statistically significant difference was observed between groups A (using non-fluoridated mouthwash) and B (using fluoridated mouthwash); therefore, the null hypothesis could not be rejected. 

Our results show that the frequency of micronuclei remained unchanged within and between the studied groups: 0–3 MN were identified corresponding to the baseline MN frequency among healthy individuals [10,29,32]. Such a result indicates the absence of a genotoxic effect of fluoride in the concentrations we used. However, a statistically significant difference in the number of cells with nuclear buds (*p* = 0.048) and karyorrhexis (*p* = 0.048) at T1 (four weeks) was observed between the A and B groups, indicating more cytogenetic damage in group B, which used fluoridated mouthwash. These results match the results of the study from Tadin et al. [1], where a significantly higher incidence of pyknotic cells and cells with karyorrhexis and nuclear buds was found by comparing kinds of toothpaste with and without fluoride and sodium lauryl sulfate. 

In 2021, two studies were published on the genotoxicity of fluoride varnishes and gels used in patients wearing fixed orthodontic appliances. Apiwantanakul and Chantaraearatit [23] found increased metal content and decreased cell viability but no genotoxic effects in patient cells of the buccal mucosa. An important limitation of their study was the Papanicolaou method of staining of buccal cells, which cannot be considered credible for the identification of micronuclei. Nersesyan et al. [33] performed comparative testing of the effects of several staining procedures on the results of micronucleus assays with exfoliated oral mucosa cells. They concluded that some nuclear anomalies and possibly keratin bodies may be misinterpreted as micronuclei with nonspecific DNA stains such as Giemsa, Papanicolaou, Diff-Quick, Azur eosin, and May–Grunwald) and lead to false positives. The use of DNA-specific stains such as Feulgen, DAPI, acridine orange, and similar dyes is mandatory for the BMCyt assay [34].

Chitra et al. [35] who also evaluated fluoride varnishes in orthodontic patients found a greater number of micronuclei at a one timepoint in the fluoridated group as compared to the non-fluoridated control. However, the authors analyzed only 200 cells per slide (per examinee) which are not enough to observe objective results. According to the original BMCyt assay protocol [32], at least 1000–2000 cells should be scored per examinee [17,29,34].

It has been proposed that fluoride can be absorbed into the oral mucosa, and that using oral hygiene products consisting of 1500 ppm of fluoride for 4 months may increase its level in the oral mucosa in humans and rat [36,37]. Studies in rats showed that repetitive and widespread use of various dental products can lead to fluoride accumulation in the oral cavity, where the excess fluoride may cause DNA damage, stimulate apoptosis, and influence the cell cycle [38,39]. 

Via application of comet assay (single-cell electrophoresis), Ribeiro et al. [40,41] demonstrated a lack of DNA damage induced by different concentrations of fluoride on mouse lymphoma cells, human fibroblasts, and rat oral cells. They concluded that NaF did not cause genotoxic alterations in rat oral cells, and that fluoride could not be genotoxic because it is not capable of forming adducts on DNA bases or intercalating in the DNA secondary structure. However, the comet assay primarily detects DNA strand breaks and other DNA lesions that are converted into strand breaks. The comet assay cannot discover fixed mutations; therefore, it does not necessarily provide full evidence of the mutagenic ability of a tested substance. 

Several other studies demonstrated the strong genotoxic nature of fluoride, but the molecular mechanisms were not completely clear [7]. It has been speculated that fluoride attacks the amine group associated with DNA directly or indirectly through free-radical production. The association of endogenous glutathione in the NaF induces genotoxicity and supports the indirect effect of fluoride on DNA by the generation of the free radicals. Jeng et al. [36] in their in vitro study concluded that NaF can be toxic to oral mucosal fibroblasts via its inhibition of protein synthesis and mitochondrial function, as well as the depletion of cellular ATP. 

Obviously, the genotoxic effect of fluoride at concentrations used in products for oral hygiene or products used in dentistry for therapeutic purposes, which are used on a daily basis, remains not fully understood and even contradictory.

Nuclear morphological alterations and different chromatin statuses have been characterized in exfoliated buccal cells as markers of cytotoxic effects; pyknosis, karyolysis, and karyorrhexis represent different degenerative and/or adaptive cellular death/apoptosis phenomena [21,29,30]. Lee et al. [3] demonstrated that 5–40 mM sodium fluoride induced apoptosis in human gingival fibroblasts through both mitochondrial and death receptor-dependent pathways. Numerous other studies have also demonstrated the effects of fluoride on apoptosis [42], cell cycle, and cell death (reviewed in Ribeiro et al. and Johnston and Strobel [21,31]).

Interestingly, we observed in this study within both A and B groups that there was a decrease in the number of binuclear cells (BNCs) from T2 to T3 and of condensed chromatin cells (CCCs) from T1 to T3. Thus, gradually increased exposure to fluoride led to a slight decrease in basal cell proliferation and apoptosis. According to Rose et al. [19], fluoride connects to oral mucosa cells mainly via extracellular calcium bridging; applying this hypothesis to the present results, the calcium-binding sites might have already been saturated by previous exposure to fluoride. 

The fact that no statistically significant fluoride genotoxicity/cytotoxicity was observed in this study may be also related to the fluoride resistance phenomenon, which was first observed in some bacterial species and strains, as well as recently in mouse cell lines and in rats (reviewed in Johnston and Strobel [31]). Rohr et al. [43] demonstrated complex global gene expression changes in mouse cells exposed to fluorides, particularly genes related to general stress response, protein synthesis, and cell membrane maintenance. Satoh et al. [44] demonstrated on three human oral cell lines (gingival fibroblasts, periodontal fibroblasts, and pulp cells) that the increase in resistance to NaF is age-related. It may be due to an increase in cellular and nuclear volumes and cellular protein content, which may result in NaF dilution near the target site. Further investigations of mechanisms of fluoride resistance will be a valuable contribution to our better understanding of the biological effects of fluoride.

The results of our study are difficult to compare with those of other studies because there is no such designed study that compared the genotoxic and cytotoxic effects on the oral mucosa using toothpaste and mouthwash together in vivo. Most of the studies found were conducted in vitro, which were quite divergent in terms of the study design features, such as the method used, type of exposure, and sample size, while the use of oral hygiene products without sodium lauryl sulfate or other well-known harmful substances may have resulted in large inconsistency among studies. 

It is important to mention that various biological, ecological, and demographic factors can influence the results of in vivo research. The oral cavity is a multifactorial environment; since each examinee has specific biological fluctuations, in vivo research is difficult to be standardized. To eradicate individual deviations, in this study, each participant served as a self-control, and interindividual biological diversity was trivial in the final assessment [22]. The prevalence of DNA damage may also be influenced by many factors. It has been shown that the MN frequency measured in cytokinesis-blocked peripheral blood lymphocytes (PBLs) using the BMCyt assay is affected by age, gender, and multiple dietary and lifestyle factors [45]. Therefore, awareness and measurement of these factors are consequently essential when performing studies of suspected exposure to genotoxic agents. In our study, meat consumption among volunteers resulted in a statistically significant positive correlation between karyolysis and gender with karyorrhexis. Ceppi et al. [11] in their review of human exfoliated buccal micronucleus assay found that the most common potential confounders are age (98.4%), gender (85.7%), and smoking habit (90.5%); however, in our study, smokers were excluded because of the possible high impact on MN frequency and generally on the results. Although the BMCyt assay has become a very popular biomonitoring tool for detecting cytogenetic damage in humans due to its simplicity and easily accessible cells, this assay also has certain critical limitations. The most important one is probably the subjective visual evaluation of slides under a microscope, where the determination of cytological parameters (endpoints) depends on the skill, training, and experience of the researcher/professionals. A study in which several laboratories from around the world evaluated the same set of slides showed a good agreement in the identification of damaged cells with micronuclei, but significant disagreement emerged regarding different endpoints of cell death. Therefore, it was recommended that anomalies associated with cell death (condensed chromatin and karyorrhectic cells) should be combined into a single category [17,43].

The present study had some limitations. The exposure to different kinds of toothpaste and mouthwash used in this experiment could require longer monitoring for the long-term biological effect, although the optimal timing to observe exfoliated cell anomalies is between 7 and 21 days, according to the basal cell turnover rate [46]. Despite the mean age of participants being similar in both groups, the cyto- and genotoxic effects may have been better investigated if all the participants were young (under 30 years old) to avoid variations depending on the rate of aging, as has been shown in recent studies on Down’s syndrome and Alzheimer’s disease [47]. A further limitation of the study was the group size [33]. Additionally, the samples were taken from the buccal mucosa only. Hence, in future studies, it should be considered to take samples from other oral mucosa locations, such as the floor of the mouth and the oropharynx, which could bring better insight into fluoride toxicity. Observing the effects of fluoridated toothpaste and mouthwash in the mouth is very complex in in vivo conditions. The main function of fluoride in oral care is to react with hydroxyapatite to form fluorapatite to enhance enamel’s acid resistance [48,49]. Considering that the dynamic competition of fluoride absorption by hard and soft tissue is unclear, these effects should also be investigated in the future. This study focused on fluoridated toothpaste and mouthwash; thus, further studies are needed to examine the potential cytogenetic damage of other oral care products. Nonetheless, our in vivo study brought useful data on the use of common fluoride oral hygiene products. Additional studies are required, comprising a higher number of examinees with a longer follow-up and maybe combining the BMCyt assay with some other methods to assess the possible DNA damage, chromosomal instability, cell death, and regenerative potential of human buccal mucosal tissue.

## 5. Conclusions

Simultaneous use of fluoride toothpaste and mouthwash regularly on a daily basis increases the amount of fluoride that comes in contact with the oral mucosa and that can be further resorbed into deeper layers of tissue and organism. This study attempted to investigate the possible harmful effects of increased fluoride amount in the oral cavity using a buccal micronucleus cytome assay. Our results showed that there was no statistically significant fluoride-dependent cytotoxic or genotoxic effect on exfoliated buccal mucosa cells for most of the endpoints of the buccal micronucleus cytome assay under the experimental conditions of this study. However, several measurements showed statistically significant discrepancies between the two study groups. Therefore, the potentially harmful cumulative effect of long-term exposure to excess fluoride originating from oral hygiene products remains open, and further research in this area with the aim of establishing safety doses is necessary and welcome. The results achieved in this paper also raise the question of the sensitivity of the BMC (BMCyt assay and suggest its supplementation with additional, more sensitive tests, preferably on a larger number of subjects.

## Figures and Tables

**Table 1:** List of ingredients in used toothpaste and mouthwashes.

Oral Hygiene Product	Manufacturer	Ingredients
**Biomed Calcimax**	Splat, Moscow, Russia	Water, hydrogenated starch hydrolysate, dicalcium phosphate dihydrate, hydrated silica, glycerin, sodium coco-sulfate, cellulose gum, sodium chloride, aroma, zinc citrate, sodium bicarbonate, benzyl alcohol, tetrasodium glutamate diacetate, *Laminaria digitata* extract, *Fucus vesiculosus* extract, *Spirulina maxima* powder extract, kaolin, xanthan gum, menthol, hydroxyapatite, arginine, menthyl lactate, *Betula verrucosa* leaf extract, *Plantago major* extract, *Thymus serpyllum* extract, *Stevia rebaudiana* extract, sodium benzoate, potassium sorbate, geraniol, linalool, d-limonene
**Toothpaste 1–0 ppm F**	Pharmagal, Split, Croatia	Water, hypermelosis, calcium carbonate, cocamidopropyl betaine, glycerol, sodium saccharin, peppermint essential oil
**Toothpaste 2–1050 ppm F**	Pharmagal, Split, Croatia	Water, hypermelosis, calcium carbonate, cocamidopropyl betaine, glycerol, sodium saccharin, peppermint essential oil, sodium fluoride (1050 ppm)
**Toothpaste 3–1450 ppm F**	Pharmagal, Split, Croatia	Water, hypermelosis, calcium carbonate, cocamidopropyl betaine, glycerol, sodium saccharin, peppermint essential oil, sodium fluoride (1450 ppm)
**Mouthwash A–0 ppm F**	Pharmagal, Split, Croatia	Water, sorbitol, glycerol, Cremophor RH 40, sodium citrate, peppermint essential oil, Cl 42090
**Mouthwash B–450 ppm F**	Pharmagal, Split, Croatia	Water, sorbitol, glycerol, Cremophor RH 40, sodium citrate, peppermint essential oil, Cl 42090, sodium fluoride (450 ppm)

**Table 2:** Baseline characteristics of the participants in study groups.

Characteristics		Group A (Mouthwash with 0 ppm F) (*n* = 20)	Group B (Mouthwash with 450 ppm F) (*n* = 21)	*p*-Value
**Age**		34.35 ± 11.61	35.67 ± 12.19	0.675
**Weight**		73.63 ± 13.16	77.06 ± 15.01	0.497
**Height**		176.35 ± 7.48	177.89 ± 7.52	0.557
**BMI**		23.44 ± 3.24	24.21 ± 3.39	0.684
**Composite fillings**		4.35 ± 3.33	6.24 ± 3.60	0.097
**Gender**	**Female**	13 (65.0%)	11 (52.3%)	0.412
	**Male**	7 (35.0%)	10 (47.6%)	

Data are presented as whole numbers and percentages or means (SD). Unpaired *t*-test for continuous values, chi-square test for categorical values; *p* ≤ 0.05.

**Table 3:** Median (interquartile range) of DNA damage parameters in buccal mucosal cells of two tested groups of participants at different timepoints.

		Group A (Mouthwash with 0 ppm F) (*n* = 20)	Group B (Mouthwash with 450 ppm F) (*n* = 21)	
	Time Point	Median (IQR)	Median (IQR)	*p*-Value
**Micronucleus**	T0—baseline (after usage of Biomed Calcimax)	0.5 (1)	0 (1)	0.880
	T1—4 weeks (after usage of Toothpaste 1 with 0 ppm F)	0 (1)	0 (1)	0.436
	T2—8 weeks (after usage of Toothpaste 2 with 1050 ppm F)	0 (1)	1 (1)	0.066
	T3—12 weeks (after usage of Toothpaste 3 with 1450 ppm F)	0 (1)	0 (1)	0.354
	*p*-value	0.888	0.471	
**Nuclear buds**	T0—baseline (after usage of Biomed Calcimax)	1 (1) ^a^	1 (2) ^b,c,d^	0672
	T1—4 weeks (after usage of Toothpaste 1 with 0 ppm F)	2 (2) ^A^	3 (7) ^A,b^	0.048 *
	T2—8 weeks (after usage of Toothpaste 2 with 1050 ppm F)	4.5 (5.5)	3 (3.5) ^c^	0.813
	T3—12 weeks (after usage of Toothpaste 3 with 1450 ppm F)	5.5 (6) ^a^	3 (4) ^d^	0.733
	*p*-value	≤0.001 *	≤0.001 *	
**Binucleated cells**	T0—baseline (after usage of Biomed Calcimax)	12 (5)	12 (9) ^f^	0.844
	T1—4 weeks (after usage of Toothpaste 1 with 0 ppm F)	12 (5)	7 (10.5)	0.210
	T2—8 weeks (after usage of Toothpaste 2 with 1050 ppm F)	14 (12.75) ^e^	10 (8.5) ^g^	0.084
	T3—12 weeks (after usage of Toothpaste 3 with 1450 ppm F)	6 (7) ^e^	6 (9.5) ^f,g^	0.813
	*p*-value	0.016 *	0.014 *	

Data are presented as the median and interquartile range. * Statistical significance was tested using the Mann–Whitney U test (between groups at the same sampling time) and Kruskal–Wallis test (within groups at different sampling times). Statistical significance was set to *p* < 0.05. The same superscript uppercase letters indicate a statistical difference between groups at the same sampling time (^A^: *p* = 0.048). The same superscript lowercase letters indicate a statistical difference within the same groups at different sampling times according to a pairwise analysis (^a–d^: *p* ≤ 0.001; ^e^: *p* = 0.020; ^f^: *p* = 0.030; ^g^: *p* = 0.043).

**Table 4:** Median (interquartile range) of cytotoxic parameters in buccal mucosal cells of two tested groups of participants at different timepoints.

		Group A (Mouthwashwith 0 ppm F) (*n* = 20)	Group B (Mouthwashwith 450 ppm F) (*n* = 21)	
	Time Point	Median (IQR)	Median (IQR)	*p*-Value
**Karyolysis**	T0—baseline (after usage of Biomed Calcimax)	180 (125)	184 (87)	1.000
	T1—4 weeks (after usage of Toothpaste 1 with 0 ppm F)	186 (86)	191 (73)	0.855
	T2—8 weeks (after usage of Toothpaste 2 with 1050 ppm F)	140 (83.25)	204 (134)	0.225
	T3—12 weeks (after usage of Toothpaste 3 with 1450 ppm F)	200 (139.75)	210 (131)	0.382
	*p*-value	0.318	0.088	
**Karyorrhexis**	T0—baseline (after usage of Biomed Calcimax)	43 (68) ^a^	40 (52) ^b,c,d^	0.990
	T1—4 weeks (after usage of Toothpaste 1 with 0 ppm F)	62.5 (61.75) ^A^	99 (56) ^A,b^	0.020 *
	T2—8 weeks (after usage of Toothpaste 2 with 1050 ppm F)	67 (66.25)	89 (53.5) ^c^	0.285
	T3—12 weeks (after usage of Toothpaste 3 with 1450 ppm F)	68 (32.25) ^B,a^	100 (50.5) ^B,d^	0.003 *
	*p*-value	0.017 *	≤0.001 *	
**Pyknosis**	T0—baseline (after usage of Biomed Calcimax)	3 (2)	2 (3)	0.926
	T1—4 weeks (after usage of Toothpaste 1 with 0 ppm F)	3.5 (2.75)	4 (2.5)	0.958
	T2—8 weeks (after usage of Toothpaste 2 with 1050 ppm F)	3 (2.5)	3 (1.5)	1.000
	T3—12 weeks (after usage of Toothpaste 3 with 1450 ppm F)	3.5 (2.75)	3 (3.5)	0.063
	*p*-value	0.182	0.402	
**Condensed chromatin**	T0—baseline (after usage of Biomed Calcimax)	20 (5.5) ^e^	20 (9.5) ^g,h^	0.927
	T1—4 weeks (after usage of Toothpaste 1 with 0 ppm F)	18 (4.75) ^f^	15 (16.5) ^i^	0.218
	T2—8 weeks (after usage of Toothpaste 2 with 1050 ppm F)	11 (10.75)	9 (4) ^g^	0.378
	T3—12 weeks (after usage of Toothpaste 3 with 1450 ppm F)	10 (10.74) ^e,f^	8 (4) ^h,i^	0.464
	*p*-value	≤0.001 *	≤0.001 *	

Data are presented as the median and interquartile range value. * Statistical significance was tested using the Mann–Whitney U test (between groups at the same sampling time) and Kruskal–Wallis test (within groups at different sampling times). Statistical significance was set to *p* < 0.05. The same superscript uppercase letters indicate a statistical difference between groups at the same sampling time (^A^: *p* = 0.020; ^B^: *p* = 0.003). The same superscript lowercase letters indicate a statistical difference within groups at different sampling times according to a pairwise analysis (^a^: *p* = 0.016; ^b,c^: *p* = 0.025; ^d–f,h^: *p* ≤ 0.001; ^g^: *p* = 0.011; ^i^: *p* = 0.008).

## Data Availability

The data presented in this study are available on request from the corresponding author.
